# An analysis of post-vocalic /s-ʃ/ neutralization in Augsburg German: evidence for a gradient sound change

**DOI:** 10.3389/fpsyg.2014.00828

**Published:** 2014-07-31

**Authors:** Véronique Bukmaier, Jonathan Harrington, Felicitas Kleber

**Affiliations:** Institute of Phonetics and Speech Processing, Ludwig-Maximilians-Universität MünchenMunich, Germany

**Keywords:** neutralization, sound change, dialect leveling, categorical vs. continuous, exemplar theory

## Abstract

The study is concerned with a sound change in progress by which a post-vocalic, pre-consonantal /s-ʃ/ contrast in the standard variety of German (SG) in words such as *west*/*wäscht* (/vɛst/~/vɛʃt/, *west/washes*) is influencing the Augsburg German (AG) variety in which they have been hitherto neutralized as /veʃt/. Two of the main issues to be considered are whether the change is necessarily categorical; and the extent to which the change affects both speech production and perception equally. For the production experiment, younger and older AG and SG speakers merged syllables of hypothetical town names to create a blend at the potential neutralization site. These results showed a trend for a progressively greater /s-ʃ/ differentiation in the order older AG, younger AG, and SG speakers. For the perception experiment, forced-choice responses were obtained from the same subjects who had participated in the production experiment to a 16-step /s-ʃ/ continuum that was embedded into two contexts: /mIst-mIʃt/ in which /s-ʃ/ are neutralized in AG and /və'mIsə/-/və'mIʃə/ in which they are not. The results from both experiments are indicative of a sound change in progress such that the neutralization is being undone under the influence of SG, but in such a way that there is a gradual shift between categories. The closer approximation of the groups on perception suggests that the sound change may be more advanced on this modality than in production. Overall, the findings are consistent with the idea that phonological contrasts are experience-based, i.e., a continuous function of the extent to which a subject is exposed to, and makes use of, the distinction and are thus compatible with exemplar models of speech.

## Introduction

The present study forms part of a series of investigations (e.g., Kleber, 2011; Müller et al., 2011; Harrington et al., 2012) into dialect leveling in High German varieties under the influence of Standard German (SG). Our particular concern is not just with phonological categorical changes in the direction of SG but more specifically with how such categorical changes are related to the continuously gradient variation in speech production and perception across generations of speakers. The present investigation deals with the association between the post-vocalic /s-ʃ/ contrast before /t/ in SG (e.g., *West*/*wäscht*; /wɛst/~/wɛʃt/, engl. *west*/*washes*) and the Augsburg variety of German (AG) in which, at least for older, but possibly not for younger speakers, the distinction is collapsed such that these minimal pairs are neutralized as a post-alveolar fricative (i.e., /wɛʃt/ for both *West* and *wäscht*). By Augsburg variety we mean a regional variety of Standard German, which is mainly influenced by the Swabian dialect.

In Standard German, the contemporary /s-ʃ/-contrast emerged as a consequence of various sound changes. Old High German (OHG) did not distinguish between those two places of articulation for fricatives, but only had alveolar sibilants, which were realized either voiceless (fortis, /s/) or voiced (lenis, /z/). The OHG /z/ later changed into the contemporary Standard German /ʃ/ (Renn and König, 2009). In addition, /s/ shifted to /ʃ/ in some /s+consonant/-clusters (/sC/ hereafter) from Middle High German (MHG) to SG. The shift from MHG /s/ to SG /ʃ/ took place only in syllable initial clusters (e.g., MHG *slagen* /slagən/ > SG *schlagen* /ʃlagən/, *to beat*), while in Southern German varieties this change also occurred in post-vocalic clusters (e.g., *fast*, engl. *almost*, which is /fast/ in SG but /faʃt/ in the south-west German variety of Swabian). However, while Bavarian (spoken in south-east Germany) nowadays contrasts /s/ and /ʃ/ before consonants just like SG, Swabian retains the pronunciation of /sC/-clusters as /ʃC/—not just in the deep dialect but also in the Swabian-colored, regional variety of Standard German. Thus, the Standard German phonemic contrast between post-vocalic, pre-consonantal /s/ and /ʃ/ is neutralized in favor of the post-alveolar pronunciation in Swabian, i.e., the minimal pair *West* (/vɛst/, *west*) and *wäscht* (/vɛʃt/, *washes*) are homophones when produced by a Swabian speaker. Nonetheless, in the Swabian variety the contrast between /s/ and /ʃ/ is maintained in intervocalic position (e.g., *Tasse* /tasə/, *cup—Tasche* /taʃə/, *bag*).

The data for the present study is taken from Augsburg—a city in Bavaria around 80 km north-west from Munich. Augsburg is situated in a transitional zone between the Bavarian and Swabian dialect areas and as a consequence, this variety has both Bavarian as well as Swabian dialect features (Nübling, 1988). In an investigation that forms the background to the present study, Bukmaier (2010) carried out an auditory analysis to determine whether the Augsburg variety should be classified as a Swabian or a Bavarian dialect based on the proportion of Bavarian and Swabian dialect features in Augsburg speakers' productions; in order to do so, she investigated the usage of dialectal features by younger (aged 20–30 years) and older (aged 40–70 years) Augsburg speakers. Her analysis showed that AG was predominantly Swabian but that there was nevertheless a tendency for younger speakers to make greater usage of SG features. It is this latter finding that is the primary motivation for the present study that focuses on the neutralization of pre-consonantal, post-vocalic /s-ʃ/ in Augsburg German.

The phonological process of neutralization is traditionally conceived as involving a categorical change from one category to another. Nevertheless, acoustic analyses have repeatedly shown that neutralization is incomplete (Port and O'Dell, 1985; Kleber et al., 2010). Similarly, the outcome of historical sound changes is usually categorical, although there is increasing evidence that a diachronic change comes about through a gradual change from one category to another across generations (e.g., Harrington et al., 2012). Since Labov's (1963) pioneering work in sociolinguistics, so-called sound changes in progress are inferred by comparing phonetic differences across two generations of the same speech community and most often within sounds that differ in continuous acoustic parameters (as the many studies on vocalic change show, e.g., Hawkins and Midlgey, 2005) since the gradual changes are perceptible and thus more obvious. There are, however, categorical sound changes such as metathesis that are typically considered to involve no such gradual change. Similarly, the auditory analysis of the data in Bukmaier (2010) points to a categorical change amongst younger speakers from AG /ʃ/ in clusters toward SG /s/.

On the other hand, research on assimilatory processes, in particular in /s#ʃ/ or /ʃ#s/ across word boundaries, has shown that sibilants vary gradually between the two places of articulation depending on the degree of assimilation (Niebuhr et al., 2008; Pouplier et al., 2011), although these fine phonetic differences may not be perceptible (Niebuhr and Meunier, 2011). Similarly, physiological studies of speech errors present evidence for gradual shifts between categories that may be perceived as clear instances of one category and may even result in auditory transcription errors (e.g., Pouplier and Hardcastle, 2005; Goldstein et al., 2007). In the light of this synchronic evidence, it seems quite possible that even these supposedly categorical diachronic changes may in fact be continuous. Thus, one of the main issues we address in this paper is whether the unmerging of /ʃt/ toward /st/ or /ʃt/ is a categorical or continuous process. A categorical change might occur lexically such that there is a discrete change for younger but not older AG speakers from /ʃt/ to /st/ in words such as West (SG /vɛst/). In a continuous change, speakers might gradually shift their production in such words between post-alveolar and alveolar productions with a greater shift toward /s/ in younger speakers.

Another major concern in this paper is whether the change affects the modalities of speech perception and production in equal measure. The arguments for parity between speech production and perception have been made across different kinds of models including at the level of gestures (e.g., Fowler et al., 2003) and also in terms of exemplar theory (Pierrehumbert, 2002) in which speech production draws upon the same sets of exemplars that have been stored in the acoustic/auditory space of the listener's mental lexicon as a result of speech perception. With respect to some sound changes, such parity can be observed within but not between generations. An example for such a sound change in progress in which there is parity between the two modalities within a generation is the age-graded neutralization of the voicing contrast of intervocalic consonants toward the lenis variant of East Franconian speakers (Müller et al., 2011). Older East Franconians neutralize the voicing contrast of Standard German plosives in perception as well as in production, while younger East Franconians neutralize this contrast equally in production as well as in perception to a lesser extent. Nevertheless, younger East Franconians do not yet maintain the voicing contrast to the same extent as Standard German speakers. The exemplar theory not only accounts for this parity but also for the shift toward the Standard German contrast^1^: the more a speaker is exposed to Standard German, the more standard forms (with all the fine phonetic detail inherent to them) are added to the edge of an exemplar cloud (i.e., the density distribution of a set of exemplars across the acoustic/auditory space that constitute a phonological category) which eventually shifts in the acoustic/auditory space and then in turn causes the speakers to select more standard-like variants from the cloud for production. On the assumption that the contact with the standard variety increases with each generation of German dialect speakers, we therefore predict with respect to the present study that younger Augsburg speakers produce sibilants before /t/ in a more standard-like way than do older speakers.

At a particular point in time during the period of change, on the other hand, sound change may also present an exceptional case in which the two modalities are out of alignment with each other (Kleber et al., 2012). According to Ohala (1981, 1993), sound change is initiated by listeners' misperceptions of speakers' production. Given the vast amount of synchronic variation in speech signals (Hawkins, 2003), misperceptions may occur under certain conditions, although these misperceptions only rarely turn into a diachronic change. A similar line of argument is found in Browman and Goldstein (1991) who present evidence for articulatory gestures that overlap to such an extent that only one gesture is decoded correctly by the listener. These forms of overlap cause at first perceptual synchronic elision, which can under certain conditions result in diachronic elision. In both models it is the mismatch between production and perception that leads to sound changes on the listener's side. Applied to the present data, AG subjects might initially unmerge /ʃt/ as /st, ʃt/ in perception with production showing a greater degree of neutralization (cf. also Labov et al., 1991).

Sound changes triggered by misperceptions of or undercompensating for synchronic variation (Harrington et al., 2008; Kleber et al., 2012) are thus driven by internal or phonetic factors. External or sociolinguistic factors such as social status or the prestige of a dialect (Kerswill, 2003; Labov, 2007) may, however, also play a role in diachronic changes—in particular those that are due to *dialect leveling*, which refers to the reduction of dialectal forms, as for example the increasing monophthongization of regional /Iə/ as /e:/ in British English with the latter having a wider geographically distribution (Kerswill, 2003). The question arises whether sound changes that are triggered by sociolinguistic factors occur passively as a result of accommodation (e.g., Trudgill, 2004) or whether the speaker takes up a more active part. The model of sound change described in Lindblom et al. (1995) emphasizes the role of the speaker to a greater extent than the above-mentioned models, as it is the speakers who adapt to listeners' needs when producing speech along a continuum from hypo- to hyper-articulated speech. Sound changes may then evolve when listeners' attention is in such circumstances exceptionally directed to a word's form (i.e., its pronunciation) instead of its meaning. Perhaps speakers of regional varieties have a propensity to evaluate the word's form when they are in contact with speakers from other varieties.

The aim of the present study was to investigate whether or not Augsburg speakers completely neutralize the /s-ʃ/-contrast in the production and perception of /sC/-clusters and whether the degree of neutralization is age-related in this variety, with younger Augsburg speakers tending to a more standard-like pronunciation. The analysis in this paper draws upon the classic technique of an apparent time investigation in which sound change is inferred by comparing phonetic differences across two generations. However, in contrast to almost all sociolinguistic investigations, the present study is based both on production and on the same speakers' responses to perceptual stimuli (see also Harrington et al., 2012, 2013; Kleber et al., 2012). The hypotheses for the two experiments can be formulated as follows:

H1: Augsburg speakers differentiate the /s-ʃ/-contrast in /st/-clusters to a lesser extent in production than Standard German speakers.

H2: Older Augsburg speakers show a greater tendency toward neutralization of the /s-ʃ/-contrast in the production of /st/-clusters than younger Augsburg speakers.

H3: Augsburg listeners differentiate the /s-ʃ/-contrast in /st/-cluster to a lesser extent in perception than Standard German speakers.

H4: Older Augsburg listeners show a greater tendency toward neutralization of the /s-ʃ/-contrast in the perception of /st/-clusters than younger Augsburg speakers.

## Production experiment

### Methods

#### Participants

The production experiment was conducted with three different subject groups: older Augsburg speakers, younger Augsburg speakers and Standard speakers. The first group—the experimental group—contained 26 speakers of Swabian from the city of Augsburg. Eleven of these subjects were aged between 40 and 70 years (3 male and 8 female) and assigned to the older age group. 15 participants were aged between 20 and 30 years (8 male and 7 female) and assigned to the younger age group. All participants were born/or have spent most of their lives in Augsburg. At the time of participation in this experiment all Augsburg subjects were living in Augsburg.

The second group served as a control group and included 16 Standard German-speaking subjects (two male and 14 female) aged between 20 and 30 years. The participants in this group were all either from Northern Germany or from Munich^2^. None of the 45 subjects reported any hearing, eye-sight, or reading problems.

Prior to the experiment the Augsburg participants were asked to fill out a questionnaire with questions about the participants education, the length of time that they had been living in Augsburg, and a self-assessment of how much and how often they speak dialect. The AG participants were chosen in accordance to the time they had been living in Augsburg; so all the young AG subjects were living in Augsburg all of their lives and the older AG participants were living in Augsburg most of their lives (30 years and more).

The subjects of the older and the younger experimental group were tested in a quiet room at their homes. The subjects of the control group were tested in a quiet room at the university. It is possible that the difference of whether the speakers were recorded at home or not could have had an influence on the results such that those recorded at home hypoarticulated more than those in the laboratory due to the slightly more informal recording setting at home. However, we found no evidence for this from our auditory impressions of the data.

#### Materials

In order to elicit productions of /st/-clusters, we designed a blending task (see also Kleber et al., 2010) in which the subjects had to combine the first syllable of one nonword with the second syllable of another nonword (see Table 1) in order to produce a real German word, e.g., the speaker's task was to produce the blend *Ki****st****e* (/kIstə/, *box*) from the two nonsense words *Kissingen* and *Wirte*.

**Table 1:** **Nonwords and resulting blends**.

**Word 1**	**Word 2**	**Blend**
Küssingen (kYsIŋən)	Wirte (/vIrtə/)	Kü**st**e (/kYstə/, *coast*)
Kissingen (kIsIŋən)	Würte (/vYrtə/)	Ki**st**e (/kIstə/, *box*)
Lüssingen (lYsIŋən)	Kirte (/kIrtə/)	Lü**st**e (/lYstə/, pl. *desire*)
Lissingen (lIsIŋən)	Kürte (/kYrtə/)	Li**st**e (/lIstə/, *list*)
Schussingen (ʃʊsIŋən)	Kirter (/kIrtɐ/)	Schu**st**er (/ʃuːstɐ/, *cobbler*)
Schwessingen (ʃvɛsIŋən)	Kürter (/kYrtɐ/)	Schwe**st**er (/ʃvɛstɐ/, *sister*)

The syllables that were blended are underlined.

With the exception of /uː/ in *Schuster*, the vowels /I/, /ɛ/, and /Y/ in the initial syllables of the resulting blends were always phonologically short, which was triggered by a word medial orthographic double consonant in the first word, e.g., <ss> in *Lü****ss****ingen* (this orthographic representation corresponds to the Standard German norm indicating phonemic short vowels). While the onset consonant varied, the coda consonant of the first syllable was always /s/. The final syllable of the second word was either /tə/ or /tɐ/ (see Table 1). The 16 filler words were disyllabic German words which did not contain any sibilants and which varied in the vowel as well as in the coda consonant of the first syllable (while the second syllable was always *−te* /tə/), e.g., *Wirte, Worte, Bunte, Kalte*.

In addition to the cluster blends, we obtained prototypical /s/ and /ʃ/ in intervocalic or post-vocalic position, i.e., in a non-neutralizing context in both varieties. For this purpose, subjects read aloud the following four German real words: *Biss* (/bIs/, *bite*), *wisse* (/vIsə/, *to know*), *Busch* (/bʊʃ/, *bush*), and *Tusche* (/tʊʃə/, *India ink*). In order to minimize any coarticulatory effects, /s/ and /ʃ/ were combined with /I/ and /ʊ/, respectively.

#### Experimental set-up, digitization, labeling

The recordings were made with the *SpeechRecorder* software (version 2.6.14; see Draxler and Jänsch, 2004), an audio interface (M-Audio Fast Track) and a stereo headset (Beyer dynamics). Each of the six target blends together with eleven distractor blends were repeated ten times and presented in randomized order on a MacBookPro computer screen (in total 170 tokens). Following the blending task, but within the same session and experimental set-up, the subjects were presented with three repetitions of each of the German real words (in total 12 tokens). In both tasks, the subjects had to produce each word within a time slot of 1 s, which was then followed by an automatic pause of 0.8 ms before the next item was presented. In total, each subject produced 182 words.

The words were digitized at 44.1 kHz. All of the data were segmented and labeled automatically into phonetic segments using the Munich Automatic Segmentation System (MAuS, Schiel, 2004); manual readjustments were made subsequently whenever necessary to the target word in PRAAT (Boersma and Weenink, 2012). All words that were mispronounced were excluded from the analysis. For the present study a total of 2996 words were analyzed, including 2494 /st/-clusters, 252 prototypical /s/ and 250 prototypical /ʃ/ (cf. Table 2).

**Table 2:** **Distribution of the 2996 /s/-/ʃ/-/st/-sequences by age group**.

	**Older AG**	**Younger AG**	**SG**
**/s/**	66	90	96
**/ʃ/**	65	89	96
**/st/**	655	883	956

#### Experimental set-up, digitization, labeling

Spectra were extracted at the temporal midpoint between each fricative's acoustic onset and offset after applying a 256 point discrete Fourier transform with a 40 Hz frequency resolution, 5 ms Blackman window, and a frame shift of 5 ms to the target words using the Emu Speech Database system (Harrington, 2010).

The subsequent parameterization of these data involved the data reduction of each spectrum (at the sibilant's acoustic temporal midpoint in all cases) to a set of mel-scaled coefficients using the discrete cosine transformation. More specifically, for an *N*-point mel-scaled spectrum, *x*(*n*), extending in frequency from *n* = 0 to *N* − 1 points over the frequency range of 500–3500 Hz, the *m*th DCT-coefficient C_*m*_ (*m* = 0, 1, 2) was calculated with the formula in (1)

(1) $$C_{m}=\frac{2k_{m}}{N}\sum_{n=0}^{N-1}x(n)\cos\left(\frac{(2n+1)m\pi }{2N}\right)$$

These three coefficients *C_m_* (*m* = 0, 1, 2) encode the mean, the slope, and curvature respectively of the signal (in this case of a given sibilant's mel-scaled spectrum extracted at its temporal midpoint) to which the DCT transformation was applied (Harrington, 2010). Since *C*_0_, which is proportional to the dB-mean across the entire spectrum, is largely irrelevant for the /s-ʃ/-distinction, only *C*_1_ and *C*_2_ (the spectral slope and curvature) were used for further quantification.

We quantified the degree of neutralization of the /s-ʃ/-distinction by calculating the Euclidean distances, *E_s_* and *E*_ʃ_, in the *C*_1_ × *C*_2_ space separately for each sibilant in the database to the Standard German speakers' /s/-centroid and to the Standard German speakers' /ʃ/-centroid, respectively. These two centroids are the positions in the *C*_1_ × C_2_ space averaged across all Standard German speakers' /s/-tokens and all Standard German speakers' /ʃ/-tokens respectively that occurred in the words from the reading condition. We then calculated for each sibilant its log-Euclidean distance ratio *d_sib_*, from (2):

(2) $$d_{sib}=\log(E_{s}/E_{\int })=\log(E_{s})-\log(E_{\int })$$

Thus, there is one *d_sib_* value per sibilant which is a relative measure: greater positive values denote a closer distance of a given sibilant to the /ʃ/-centroid; greater negative values are associated with distances closer to the /s/-centroid; and a value of zero on *d_sib_* denotes that a given sibilant is equidistant in the *C*_1_ × *C*_2_ space between the /s/ and /ʃ/-centroids (e.g., Harrington et al., 2008; Kleber et al., 2012, for a similar methodology).

### Results

**Figure 1:**
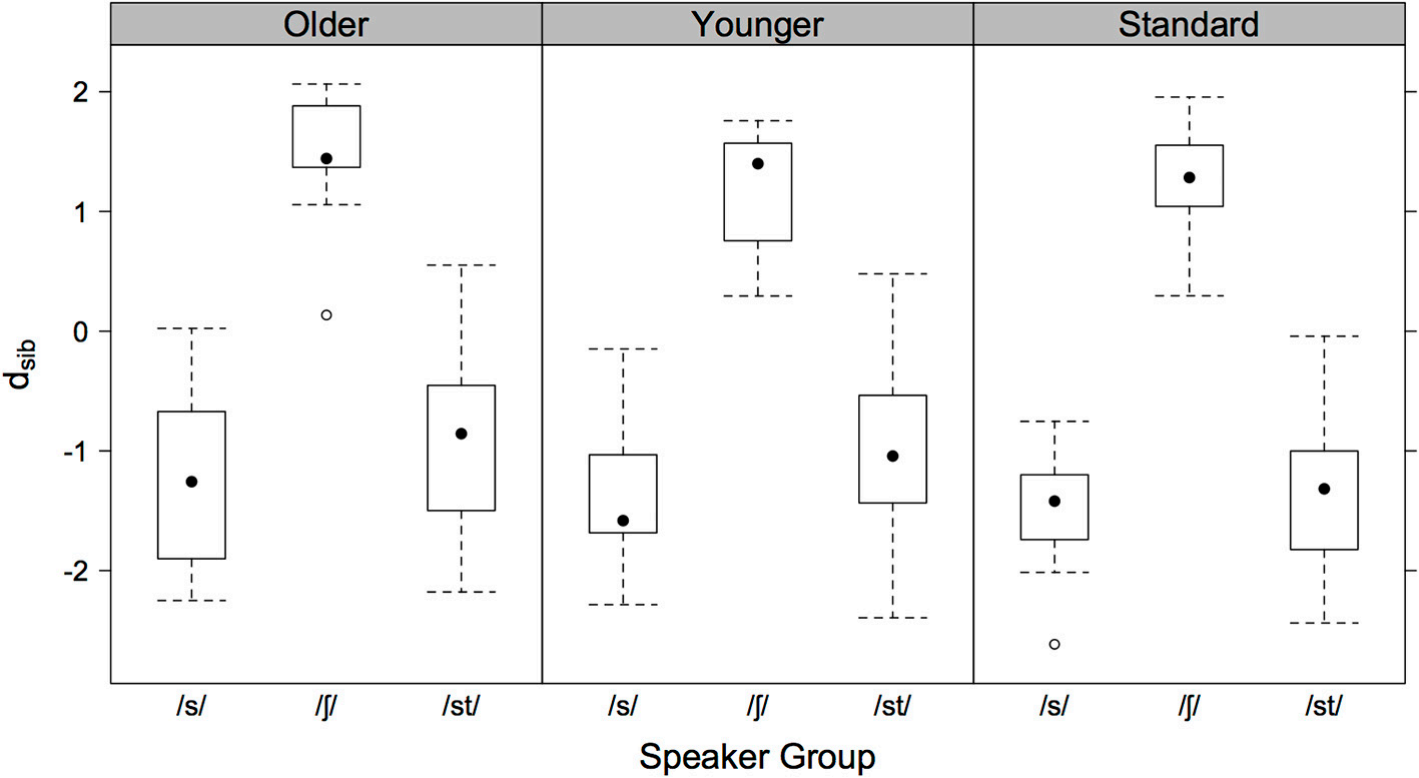
**Boxplots of the log**. Euclidean distance ratio, *d_sib_* for older AG (left), younger AG (center), and SG speakers (right). Negative values indicate productions closer to the /s/-centroid, positive values are productions closer to the /ʃ/-centroid.

Figure 1 shows for each speaker group the log-Euclidean distance ratio, *d_sib_*, for their singleton and cluster sibilants to the /s/ and /ʃ/-centroids. Negative/positive values are productions of a given sibilant closer to the /s/ and /ʃ/-centroids respectively. As Figure 1 shows, all speaker groups produced cluster sibilants as more /s/-like, although those of older and younger AG speakers tended to be closer to the /ʃ/-centroid than those of the SG speakers: this is evident in the medians (the dots in Figure 1) which are higher (closer to zero) in /st/ for AG than for SG speakers.

**Figure 2:**
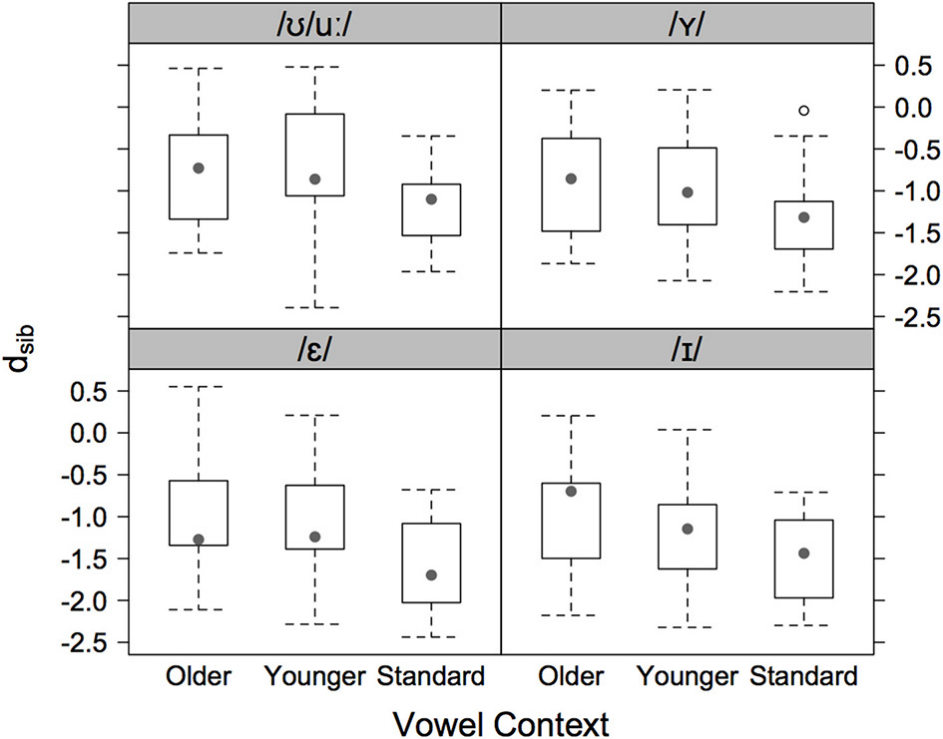
**Boxplots of the log**. Euclidean distance ratio, *d_sib_* for sibilants in /st/-clusters to the /s/ and /ʃ/-centroids (older AG left, younger AG center, and SG right) and vowel context (/ε/ bottom left, /I/ bottom right, /Y/ top right, /ʊ/ top left). Negative values indicate productions closer to the /s/-centroid, positive values are productions closer to the /ʃ/-centroid.

Figure 2 shows separately for each speaker group and vowel context (/ε I Y ʊ u:/) *d_sib_* for the sibilants in /st/-clusters to the /s/ and /ʃ/-centroids. In these data, older AG speakers have values closest to zero: this shows that their productions were slightly more /ʃ/-like than for the other two groups. At the same time, the SG speakers always had the lowest median values such that their /st/ was closest to /s/ compared with the AG speakers. Figure 2 also shows that the younger AG speakers' medians were between those of the other two groups. A mixed model with *d_sib_* (the data in Figure 2) as the dependent variable and with vowel context (/ε I Y ʊ u:/) and speaker group coded for increasing order (three ordered levels: older Augsburg > younger Augsburg > Standard) and with speaker as the random factor showed a significant effect for vowel [χ^2^_(1)_ = 30.4, *p* < 0.001], a significant effect for group [χ^2^_(1)_ = 4.7, *p* < 0.05], and no interaction between these factors. The significant effect for group is a confirmation of the evidence in Figure 2 that there is a trend from older AG to younger AG to SG speakers for /st/ to be progressively closer to /s/.

## Perception experiment

### Methods

#### Participants

The participants were the same as in the production experiment. The production and perception experiments were both run in one session per speaker (always starting with the production experiment), i.e., each subject who had participated in the production experiment completed the perception experiment as well.

In order to control for the effect of biological age (i.e., for differences between groups that are not due to the dialectal background but that might come about because of an age-related diminished capacity for identifying high-frequencies that are critical for place of articulation distinctions in fricatives), we included a fourth subject group consisting of older (aged between 40 and 70 years; 7 males and 8 females) Standard German listeners. The older SG listeners were born and lived in Northern Germany (near the city of Hannover). They were tested in a quiet room at their homes. None of them reported any hearing, eye-sight, or reading problems.

#### Materials

For the perception experiment, we created two synthetic continua between /s/ and /ʃ/ using STRAIGHT Tandem (Kawahara et al., 2008). The first continuum extended between the minimal pair *Mist* (/mIst/, *dung*) and *mischt* (/mIʃt/, *mix*). In this context, we expected AG listeners to have difficulty perceiving the contrast, given the tendency to produce both words as homophones in this variety (we will henceforth refer to this continuum as the *ambiguous* context). The second continuum (the unambiguous context) extended between *vermisse* (/və'mIsə/, first pers. sing. *miss*) and *vermische* (/və'mIʃə/, first pers. sing. *mix*). For this continuum, we expected no difference between the groups, since the /s-ʃ/-contrast is contrastively produced in both Augsburg and Standard German.

Both continua were derived from natural productions of *vermisse* and *vermische* spoken by a Standard German speaking phonetician. We recorded several repetitions of these two words and selected two prototypical realizations. These two selected /və'mIsə/ and /və'mIʃə/ sound files were morphed by adding time anchors to the /s/ and /ʃ/-sequences and setting frequency anchors for the added time anchors. This was done to get a horizontal overlap between the two sibilants. After creating a 22-step continuum between /və'mIsə/ and /və'mIʃə/ the mi[s/ʃ]-sequence was cut out of the created continuum. We then selected stimuli 1, 3, 5, 7, 8, 9, 10, 11, 12, 13, 14, 15, 16, 18, 20, 22 (i.e., selected only 16 stimuli from the original 22 steps continuum^3^) for our perception experiment. After we had selected the stimuli, we prepended the synthetic mi[s/ʃ]-sequences to a following −*t* (to create the ambiguous continuum *Mist*-*mischt*) and spliced the same synthetic sequences between *ver___e*, (to create the unambiguous continuum *vermisse-vermische*).

#### Experimental procedures

The perception experiment was conducted using *Praat's ExperimentMFCscript.* Listeners judged all 320 stimuli (16 stimuli × 10 repetitions × 2 contexts) in a two-alternative forced-choice identification task. The order of presenting the continua was counterbalanced, i.e., some subjects first listened to the /mIst/—/mIʃt/ continuum and afterwards listened to the /və'mIsə/—/və'mIʃə/ continuum and vice versa. All stimuli were presented to the listeners over headphones. Upon presentation of an auditory stimulus, the subject saw an orthographic representation corresponding to the minimal pair distinction. For example, the subject heard a stimulus from the /və'mIsə/—/və'mIʃə/ continuum and saw *vermisse* and *vermische* on the screen. The task then was to judge whether the stimulus sounded more like *vermisse* or *vermische*. The order of the stimuli was random for each participant to avoid any presentation effects. The experiment was self-paced, i.e., the next stimulus was only presented after the subject had made a decision and after a stimulus initial silence of 0.5 s. The perception experiment took about 20 min per listener.

#### Data analysis

**Figure 3:**
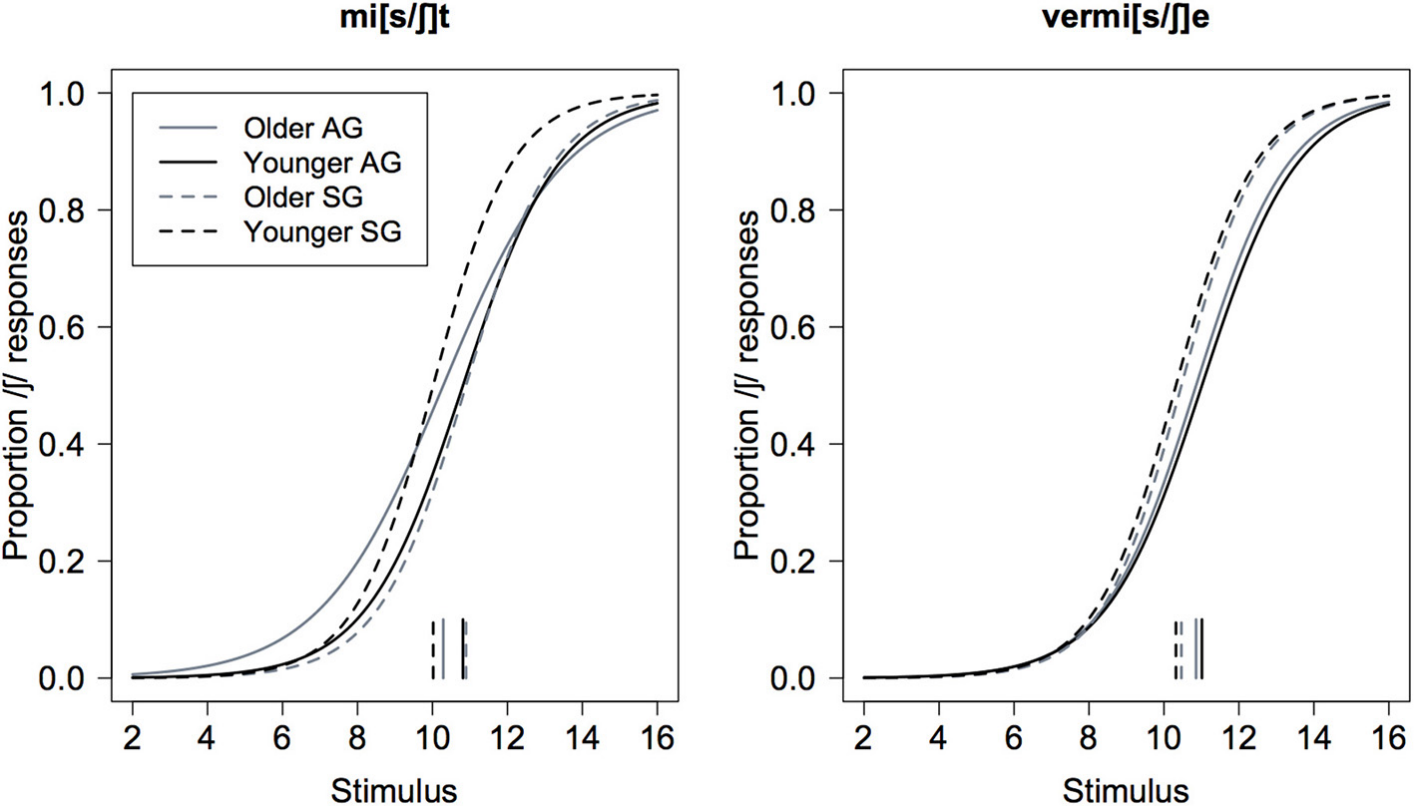
**Psychometric curves of the proportion of /ʃ/ responses as a function of stimulus number for the mi[s/ʃ]t (left) and vermi[s/ʃ]e (right) continua shown separately for AG (solid) and SG (dashed) listeners, and for older (gray) and younger (black) listeners**. The vertical lines at the bottom of the display are the decision boundaries for which the /s/ and /ʃ/ responses are equiprobable (and equal to 0.5).

We fitted eight logistic regression models to the responses, one for each of the possible combinations of age (younger vs. older), variety (AG vs. SG), and continuum-type (mi[s/ʃ]t vs. vermi[s/ʃ]e). For each of these 8 models, the dependent variable was the binary responses (/s/ or /ʃ/), and the integer stimulus number (1 ≤ *n* ≤ 16) was the independent (numerical) factor. The output of this analysis was used to derive psychometric curves separately by age, variety, and continuum type (Figure 3 below).

We then re-ran the same 8 logistic models, but this time included for each of them an interaction term between the stimulus and the listener: with this technique, we derived slopes, intercepts, and decision boundaries for each listener. All of the listener-specific decision boundaries fell within the range of the stimuli (i.e., between 1 and 16). However, the data from one younger AG listener on the mi[s/ʃ]t continuum and from one older Standard listener on the vermi[s/ʃ]e continuum were subsequently excluded from any further analyses because their slopes could not be unambiguously determined^4^.

### Results

As the short vertical lines at the bottom of Figure 3 show, there seems to be no systematic influence of any of the main factors on the decision boundary (the point at which the probability of /s/ or /ʃ/ responses are equal and 0.5). On the other hand, they do have an influence on slope: in particular, the slope is clearly steeper (i.e., the psychometric functions have a more pronounced sigmoid-shape) for the Standard vs. the Augsburg listeners in both continua. In addition, the same figure suggests that there may be a steeper slope for the younger vs. older Augsburg listeners in the mi[s/ʃ]t than in the vermi[s/ʃ]e continuum (compare the solid black with the solid gray curves in the left panel of Figure 3).

**Figure 4:**
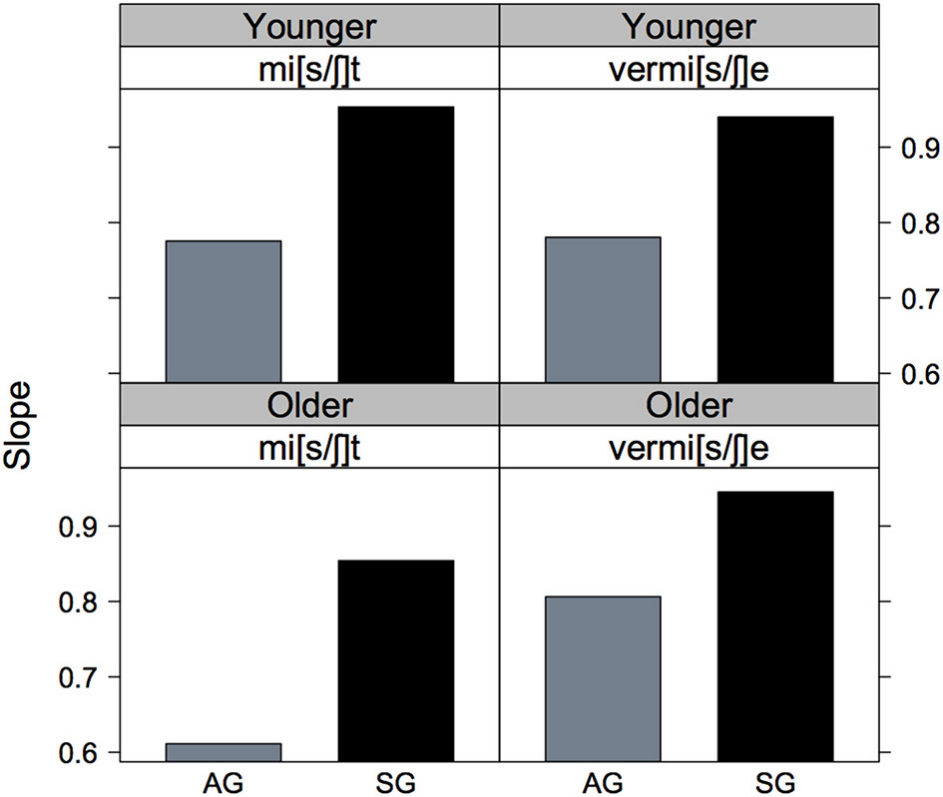
**The slopes of the psychometric curves shown in Figure 3 for mi[s/ʃ]t (left column) and vermi[s/ʃ]e (right column) continua and for younger (row 1) and older (row 2), AG (gray) and SG (black) listeners**.

The barchart of these eight slope values in Figure 4 shows more clearly the steeper slopes for Standard vs. Augsburg listeners in all cases, as well as the steeper slope for the younger than for the older Augsburg listeners in the mi[s/ʃ]t vs. vermi[s/ʃ]e continuum.

**Figure 5:**
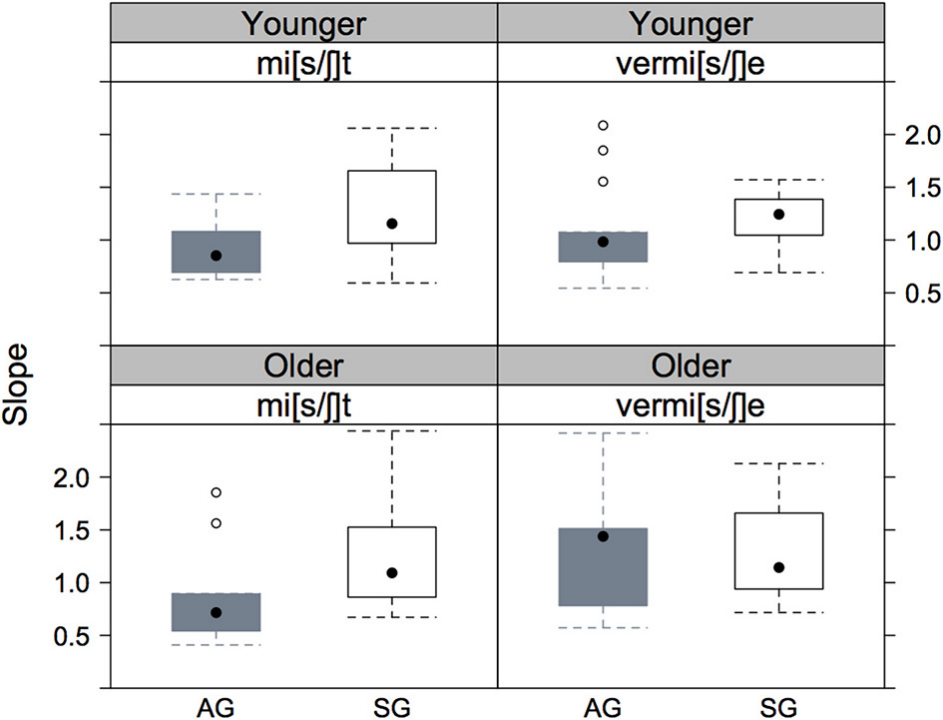
**The distribution of the listener-specific slopes on the three fixed factors**. There is one point per listener in each distribution. The rectangle spans the inter-quartile range; the black dot in the center of the rectangle is the distribution's median.

Figure 5 of the listener-specific slopes shows only some of the trends that were apparent from the analyses based on the entire population of listeners in Figures 3, 4. The clearest consistency is in the effect of variety: with the possible exception of older listeners on the vermi[s/ʃ]e continuum, there is strong evidence that the slopes are steeper for the SG compared with the AG listeners. However, a comparison of the first with the second row does not confirm the earlier observation of any influence of age group on slopes.

In order to quantify these observations further, we ran a mixed model with slope as the dependent variable, with the listener as a random factor, and with three fixed factors: age (older vs. younger), variety (AG vs. SG), and continuum-type (mi[s/ʃ]t vs. vermi[s/ʃ]e). The results (see also Table 3) showed significant main effects for variety [χ^2^_(5)_ = 6.3, *p* < 0.05] and for continuum-type [χ^2^ = 5.0, *p* < 0.05], but no effect for age group^5^. The results also showed no significant interactions between any of the fixed factors.

**Table 3:** **Estimates, Standard error, and t-statistics for the independent factors in the mixed model with slope as the dependent variable**.

**Factor**	**Estimate**	**St. Error**	***t*-value**
Continuum	0.41	0.15	2.82
Age	0.06	0.19	0.34
Dialect	0.42	0.19	2.22
Continuum × Age	−0.27	0.20	−1.37
Continuum × Variety	−0.35	0.20	−1.80
Age × Variety	−0.08	0.26	−0.31
Continuum × Age × Variety	0.26	0.26	0.99

**Figure 6:**
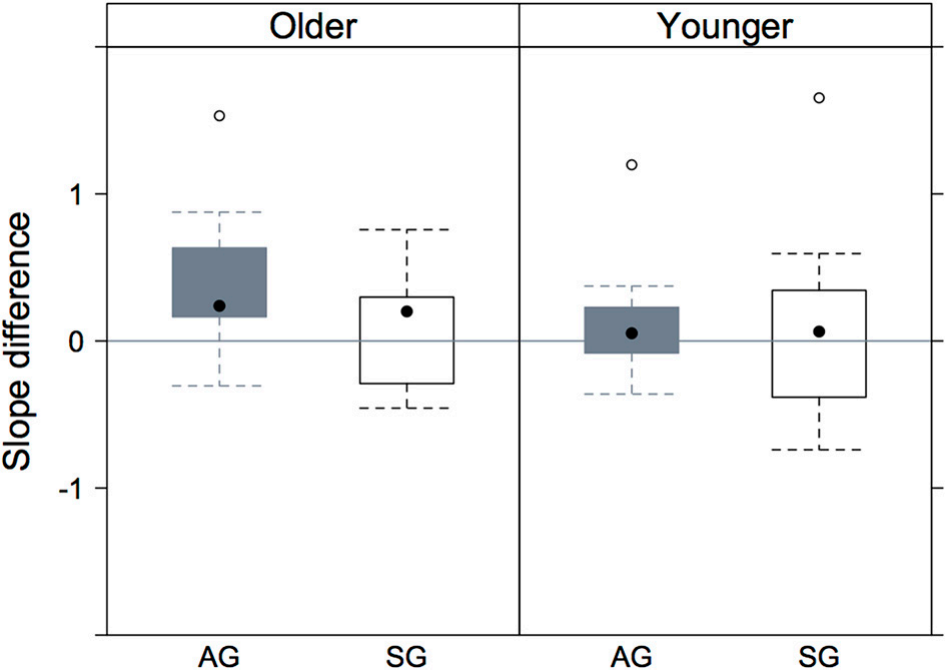
**The distributions resulting from subtracting the slopes of the mi[s/ʃ]t from those of the vermi[s/ʃ]e continua separately per listener**. There is one point per listener in each distribution. The rectangle spans the inter-quartile range; the black dot in the center of the rectangle is the distribution's median. Values above the horizontal zero line denote a larger slope in vermi[s/ʃ]e than in mi[s/ʃ]t.

The significant effect of continuum type is to a certain extent evident in Figure 6 in which the slopes in the vermi[s/ʃ]e continuum have been subtracted from those in the mi[s/ʃ]t continuum separately per listener. The null hypothesis is that the two continua do not differ on slope in which case the difference between the continua in Figure 6 on slope should be zero. Figure 6 shows that the median of all four distributions is above zero which means that, compatibly with the statistical analysis, the slopes were steeper on the vermi[s/ʃ]e than on the mi[s/ʃ]t continuum. Additionally, there was a trend for greater slope differences between continua in older Augsburg listeners (as opposed to all other speaker groups) since it is only for this group that the lower quartile is above zero.

**Figure 7:**
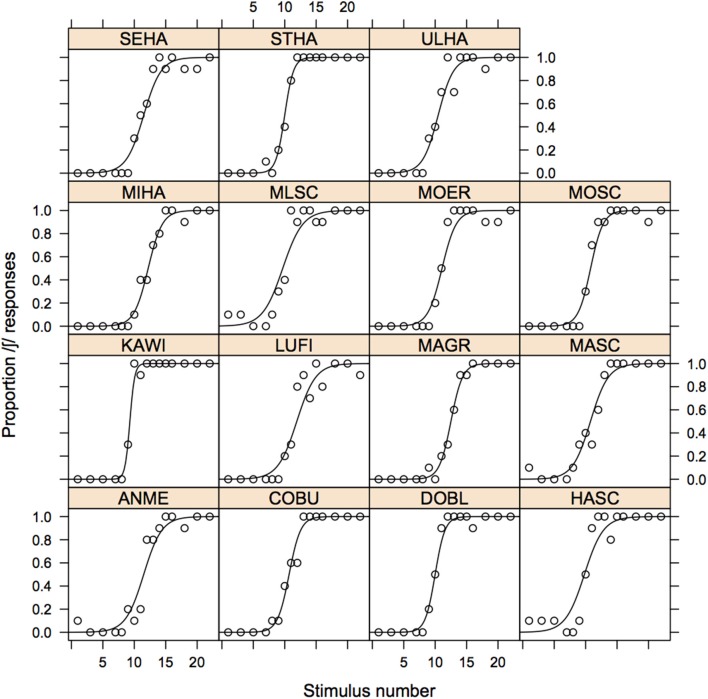
**Psychometric curves of the proportion of /ʃ/-responses for the younger Augsburg group to the stimulus of the mi[s/ʃ]t continuum shown separately for each listener together with the raw values shown as data points**.

## Discussion

The aims of the present study were two-fold: the first was to investigate a potential sound change in progress in the Augsburg variety of German and the second to examine whether an apparently categorical sound change is gradual across generations. The motivation for this study was Bukmaier's (2010) analysis showing evidence that younger Augsburg speakers use less dialectal features such as /ʃt/ instead of Standard German /st/ than older Augsburg speakers. To address the present research questions, analyses of both production and perception data were mandatory. There are three main findings from this production and perception study, which are discussed below.

The first finding comes from the analysis of the production data showing that—although both Augsburg and Standard German speakers maintained the /s-ʃ/-contrast before /t/ and produced the fricative as /s/ in this position—the sibilant in the cluster was further away from /s/ for AG compared with SG speakers. Thus, this finding supports hypothesis H1 according to which Augsburg speakers maintain the /s-ʃ/-contrast to a lesser extent in the cluster context than do Standard German speakers. As far as speaker age is concerned, hypothesis H2 predicted that the /st/-productions of younger Augsburg speakers should be between those of the older Augsburg and the Standard speakers. Our results were consistent with this hypothesis. Younger Augsburg speakers' sibilants were more /s/-like than those of their older counterparts, but not as /s/-like as those of the Standard speakers.

According to hypothesis H3, the /s-ʃ/-neutralization in production in Augsburg German should have an impact on perception: that is, the /s-ʃ/-contrast should not be as perceptually distinctive for AG as for SG listeners. Based on this hypothesis, we predicted that Augsburg subjects would perceive more instances of the /st-ʃt/-continuum as /ʃt/ with the category boundary either shifted toward the /s/-end of the continuum or even with no shift from /ʃ/ to /s/ in case of (in)complete neutralization of the contrast. Our results provide partial support for H3. On the one hand, the location of the /s-ʃ/-category boundary was similar for the three groups, suggesting that there is no preference for AG listeners to perceive /ʃ/, even though /ʃt/ is much more frequent in the Augsburg dialect than /st/. This result does not match Kleber et al.'s (2010) findings showing a bias in listeners' responses toward sound sequences that occur more often in a variety that the speaker is frequently exposed to. On the other hand, the results from the slopes were consistent with our hypothesis: the flatter slopes for the AG listeners are consistent with the idea that there is a greater ambiguity for AG than for SG listeners in categorizing an /s-ʃ/-continuum: that is, Augsburg listeners perceived the contrast less sharply than SG listeners.

The age^6^ effect too was less apparent in the perception than in the production data. While younger listeners' response curves appeared to be steeper and thus more categorical, this observation did not reach significance when taking differences between individuals within a speaker group into account. Therefore, hypothesis H4, which predicted that older Augsburg listeners should perceive this contrast to a lesser extent than younger Augsburg listeners is not quite supported. The results showing greater similarities across the three groups in perception than in production may be consistent with the idea that the sound change is more advanced in perception than in production. This is compatible with other findings showing a potential misalignment between the two modalities during a sound change in progress such that perception precedes production (Ohala, 1993; Kleber et al., 2012). Thus, for the present data, while older AG subjects are the most conservative of the three groups in production (because their sibilants are closest to /ʃ/), they are similar to the younger AG listeners in how they cut up the /s-ʃ/-continuum in perception. Despite the nonsignificant age-effect, our data is consistent with the view that younger speakers lead this sound change in progress from /ʃt/ to /st/ (Labov, 2007) since in older as opposed to younger participants' data there was (1) a trend toward flatter /st-ʃt/-curves (2) an apparently greater slope difference between the vermi[s/ʃ]e and the mi[s/ʃ]t continua, and (3) a more /ʃ/-like pronunciation of the cluster sibilant.

In general, the Augsburg participants maintained the contrast both in perception and production in a categorical manner and thus surprisingly well. This result is probably due to Augsburg participants' awareness of the contrast between the sibilants before stops in SG. The awareness comes about because (1) they learn the standard realization in school, (2) the Augsburg variety has a phonemic /s-ʃ/-contrast in intervocalic position, and (3) because they are of course exposed to the Standard German variety. Speakers also often target a standard pronunciation in a laboratory recording session. Knowledge of the contrast facilitates its production even if /ʃt/ is characteristic of their variety. For example, Broersma (2005) found that Dutch listeners' performance in perceiving the final voicing contrast in English words was similar to those of English native speakers even though the voicing contrast is neutralized in final position in Dutch. She explained this finding with the listeners' capability of transferring perceptual cues from a contrast in a familiar position (such as the voicing contrast in intervocalic position in Dutch) to the same contrast in an unfamiliar position. The results from her study are consistent with our findings in perception. In addition, our production data show that speakers may also transfer these cues to the production of a contrast in an unfamiliar position—even in a blending task that is designed to obscure the aim of a study and to prevent hyperarticulation.

Studies based entirely on auditory impressions and transcriptions are not suitable for detecting the subtle differences between speaker groups observed in the present study. It is the dialect- and age-grading found in the acoustic analyses and to a certain extent in the perception data which shows that the transfer of standard forms to the Augsburg variety is not a categorical change from /s/ to /ʃ/. Thus, the third important finding from this study is that sounds that give the auditory impression of a categorical change may nevertheless show remnants of the old or dialectal form in the acoustic signal. That is, traces of a gradual shift from the old variant toward the new variant are still present. In this respect our results are consistent with findings from physiological studies showing that articulatory traces from a segment may still be observable even though the segment is not perceptible (Pouplier and Hardcastle, 2005; Pouplier, 2007).

The findings from speech production and perception are in general consistent with previous results on gradual sound changes in German regional varieties that are most likely to evolve under the influence of the standard variety and thus can be regarded as a form of dialect leveling (Kerswill, 2003). For example, Kleber (2011) showed that, while the long/short vowel contrast tends to be neutralized before fortis stops in older Bavarian speakers, such a contrast is beginning to develop toward that of the standard variety for younger Bavarian speakers. Müller et al. (2011) report a gradual change from dialectal lenis stops toward standard fortis stops from older to younger East Franconian speakers. Similarly to East Franconian, fortis stops are lenited in Upper Saxon. Although Kleber (in press) found no age-grading in her Saxon data, she argues that the sound change in progress may be more advanced in Saxon as both older and younger Saxon speakers behaved like younger East Franconian speakers and listeners in a study by Müller et al. (2011). In addition, there was a trend toward flatter psychometric curves derived from the older Saxon listeners to a fortis-lenis continuum—similar to the data presented in this study. These forms of dialect leveling are very likely to come about because of the speakers' increasing contact with the standard language—for example, in school (Besch, 1983), via the media (cf. Stuart-Smith et al., 2013) and generally as a result of higher speaker mobility (cf. Clopper and Pisoni, 2006). The position of Augsburg in a transitional zone between Swabian and Bavarian (with Bavarian speakers patterning with Standard speakers in relation to the /s-ʃ/-distinction) might further strengthen the influence of the standard variety on Augsburg German.

These forms of gradual sound changes as a consequence of dialect leveling are best explained in a usage-based model of speech perception such as the exemplar theory of speech perception (Johnson, 1997; Pierrehumbert, 2002) according to which each perceived token with all its fine phonetic detail is added to the neighborhood of the most similar exemplar in an acoustic-perceptual space of the listener's mental lexicon and where phonological categories emerge from the density distribution of the stored exemplars. The resulting exemplar cloud is not fixed to a certain point in this acoustic/auditory space, but it may shift as new exemplars that differ slightly in their acoustic make-up from the other exemplars in this cloud are added. The probability of a shift in the exemplar cloud is increased when more and more variants with properties that are auditorily at the edges of the cloud are added. Thus, in terms of this model, a shift toward the standard variety may be caused by a progression of the cloud as more pre-consonantal /s/-exemplars from the standard variety are stored: the greater shift observed in younger AG subjects is because they are, or have been, exposed to a greater extent to SG than older subjects.

The conclusion so far is that external factors (Kerswill, 2003) cause sound change that can be associated with a general trend of dialect leveling in German regional varieties. External factors may influence sounds such that they change in a direction that would not have been predicted by phonetically motivated internal factors (Torgersen and Kerswill, 2004; Kleber, 2011; Müller et al., 2011). The present sound change in progress, however, may not only be driven by variety contact but also by internal phonetic factors. The phonetic basis of a diachronic change from /ʃ/ to /s/ before /t/ lies in the generally higher spectral peak in /ʃ/ before /t/ due to the coarticulatory effect of the alveolar stop thus pushing the sibilant toward an alveolar place of articulation. This may also explain the finding of slightly steeper vermi[s/ʃ]e than mi[s/ʃ]t-curves in all speaker groups including in the standard group (cf. Figure 6). That is, standard listeners are more variable in their choices between /s/ and /ʃ/ in an mi[s/ʃ]t-continuum because of the greater ambiguity in deciding whether a higher spectral peak is a property of the fricative itself or instead caused by the coarticulatory influence of the stop's alveolar place of articulation. Such a perceptual account would explain why a diachronic change from /s/ to /ʃ/ before /t/ is much more likely than a change from /ʃ/ to /s/.

To conclude, our findings provide evidence for a sound change in progress that affects both perception and production and which is primarily the result of the external influence of the standard variety on Augsburg German. This type of sound change patterns with a more general trend of dialect leveling in German regional varieties. Together with the results of previous studies on regional varieties of German such as East Franconian (Müller et al., 2011; Harrington et al., 2012), Saxon (Kleber, 2011, in press) and Bavarian (Kleber, 2011), these findings support the idea that the shift from one phonological category to another is gradual rather than abrupt in a context in which the categories are neutralized. In this respect, our results contribute to the longstanding debate on whether sound changes are categorical or whether phonological processes such as neutralization are complete. Phonological categories such as voiced vs. voiceless or (as in the present study) alveolar vs. post-alveolar mark endpoints of phonetic continua that span not only hyper- or hypoarticulated forms but also other forms of indeterminacy such as incomplete neutralization. Speakers produce and perceive variants along these continua. Diachronic changes may then come about when the distribution of variants along the continuum is incrementally shifted due to external factors. This idea is compatible with usage-based theories of speech perception as well as theories in which perception leads production during a sound change in progress. Future research is necessary to probe more deeply the mechanisms underlying diachronic change by investigating, for example, whether gender and social class differences or gradual shifts along phonetic continua are reinforced in certain conditions such as different prosodic contexts or speech rates, and in certain age groups, e.g., in children during phonological acquisition.

### Conflict of interest statement

The authors declare that the research was conducted in the absence of any commercial or financial relationships that could be construed as a potential conflict of interest.

## Footnotes

^1^The direction of this change is not easily accounted for by other phonetic models of sound change as phonetically lenition is much more likely to occur than fortition as the many diachronic lenitions in Romance languages show.

^2^The dialect spoken in Munich is not affected by the dialect feature in this study, i.e., the Munich variety has exactly same /s-ʃ/ contrast distribution as Standard German.

^3^In order to create a continuum with a finer separation between stimuli in the middle of the continuum (i.e. the ambiguous part) we morphed a 22-step continuum. Since this fine separation was not necessary for responses to stimuli at the edges of the continuum, we discarded every second stimulus at the beginning and end of the continuum; another reason for discarding some stimuli was to shorten the duration of the perception experiment.

^4^This comes about for the type of data exemplified by listener KAWI in Figure 7 for which there is only a single point (at stimulus 9) in the region of ambiguity between /s/ and /ʃ/ responses as a result of which the slope cannot be unambiguously determined (giving rise to the error message in R “fitted probabilities numerically 0 or 1 occurred”).

^5^We included a term for random intercepts for speakers which quantifies by-speaker variability in the dependent variable *d_sib_*. This was because there were insufficient tokens for convergence to be obtained by additionally including random slopes. We applied a repeated measures ANOVA to the same data, in order to assess the validity of the mixed model with random intercepts only. The results showed significant influences on slope of continuum [*F*(1, 51) = 5.8, *p* < 0.05] and of variety [*F*(1, 51) = 6.4, *p*< 0.05] but not of age, and there were no significant interactions. These results are entirely consistent with those obtained from the intercept only mixed model (and also comparable in the F-statistic size and probabilities for the significant results for continuum and variety).

^6^Age as an artifact due to the potentially diminished perceptual capacities in older listeners can be ruled out as there were no significant differences between younger and older listeners in the standard group. That means that older speakers did not perform in general worse in sibilant perception than younger listeners.
